# Identification and Endodontic Management of Middle Mesial Canal in Mandibular Second Molar Using Cone Beam Computed Tomography

**DOI:** 10.1155/2015/867976

**Published:** 2015-11-17

**Authors:** Bonny Paul, Kavita Dube

**Affiliations:** Department of Conservative Dentistry and Endodontics, Hitkarini Dental College and Hospital, Jabalpur, Madhya Pradesh 482005, India

## Abstract

Endodontic treatments are routinely done with the help of radiographs. However, radiographs represent only a two-dimensional image of an object. Failure to identify aberrant anatomy can lead to endodontic failure. This case report presents the use of three-dimensional imaging with cone beam computed tomography (CBCT) as an adjunct to digital radiography in identification and management of mandibular second molar with three mesial canals.

## 1. Introduction

The success of any endodontic treatment depends upon a number factors, namely, thorough knowledge of internal anatomy of the root and its canals, thorough knowledge of instrumentation techniques, and thorough cleaning, shaping, and filling of the canals. Studies on the internal and external anatomy of teeth have shown that anatomic variations can occur in all groups of teeth and can be extremely complex. Therefore it is imperative that aberrant anatomy be identified prior to and during root canal treatment of teeth.

Literature mentions mandibular second molars as teeth usually having two roots and three root canals. However variations from these have been reported in various studies [1, 2]. Aberrations such as C shaped canal systems have been reported in mandibular second molars [3, 4]. A middle mesial canal is sometimes present in the developmental groove between mesiobuccal and mesiolingual canal of mandibular first molar and the incidence ranges from 1 to 15% [5]. This additional canal may have a separate foramen or may join apically with either mesiobuccal or mesiolingual canal. However middle mesial canals are more common in mandibular first molars and have rarely been reported in mandibular second molars [6, 7].

This paper reviews the literature about middle mesial canal in mandibular second molars and reports a case of middle mesial canal using CBCT imaging effectively.

## 2. Case Report

**Figure 1:**
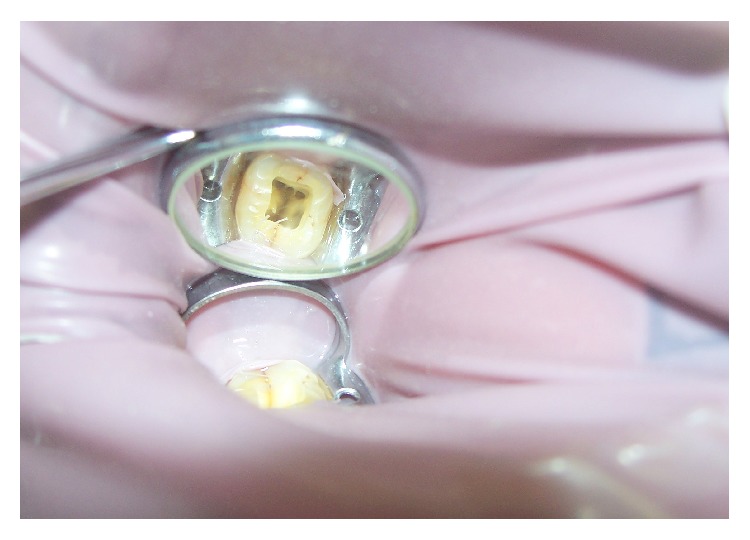
Access opening.

**Figure 2:**
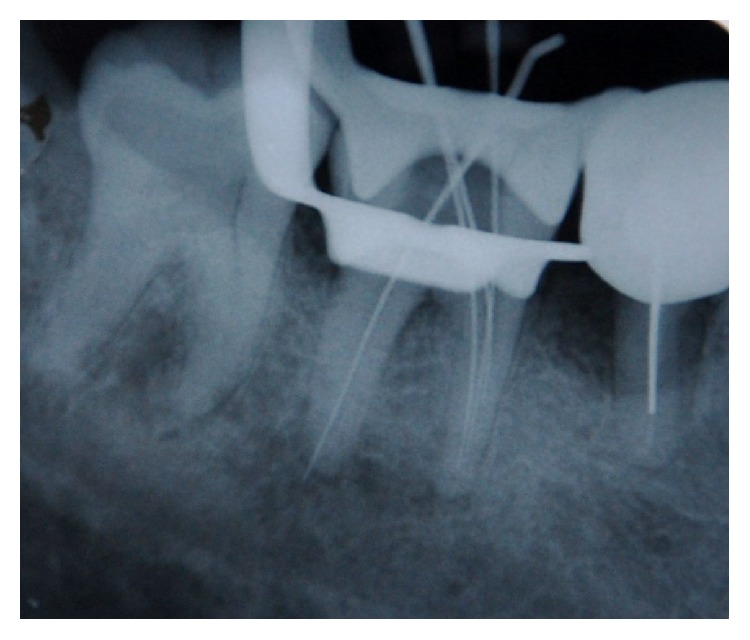
Working length.

**Figure 3:**
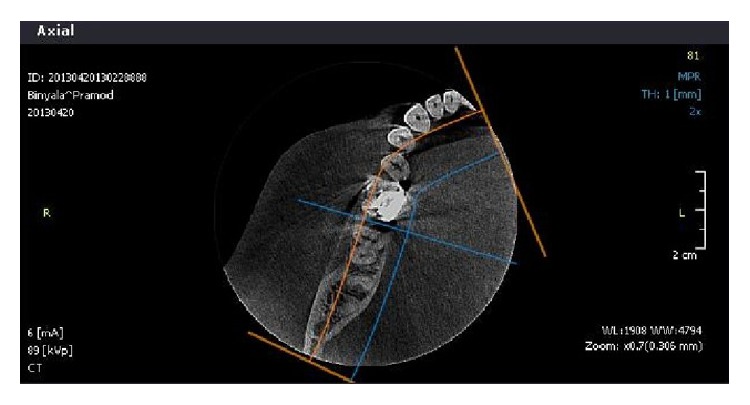
CBCT.

**Figure 4:**
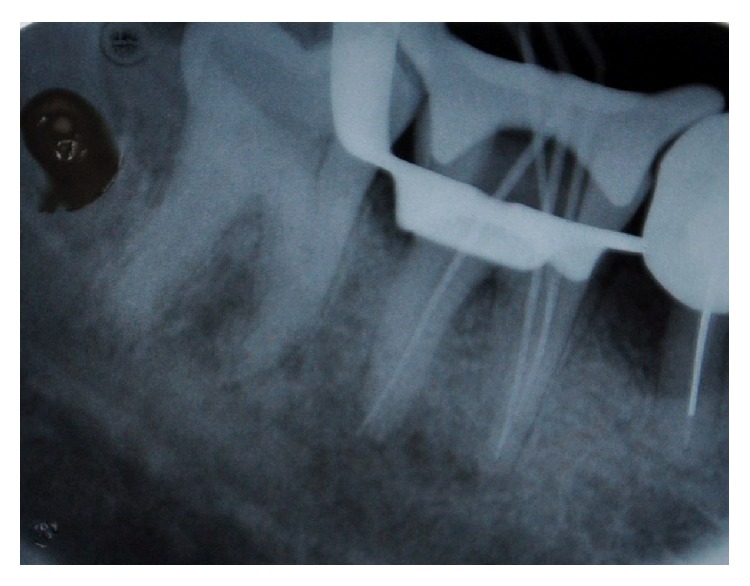
Master cone.

**Figure 5:**
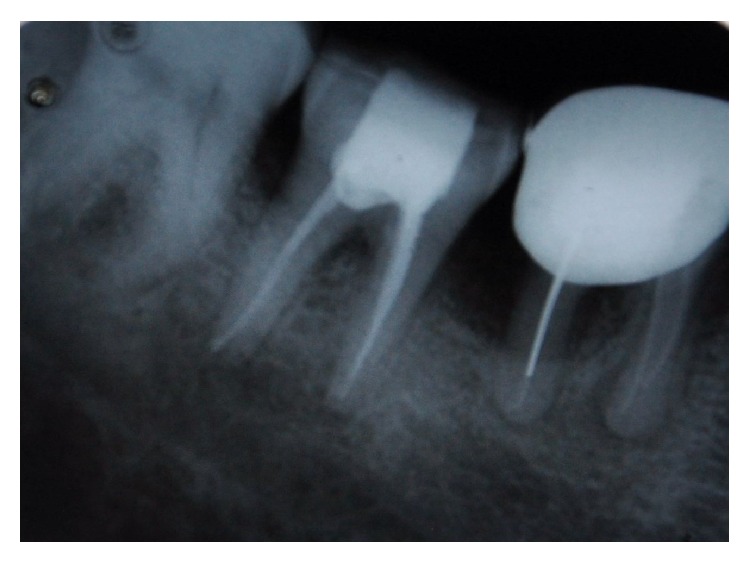
Obturation.

**Figure 6:**
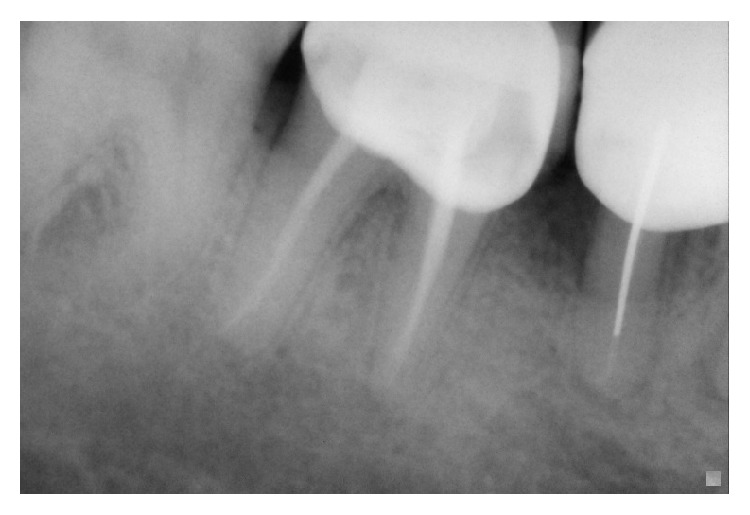
Follow-up radiograph.

A 45-year-old patient with a noncontributory medical history reported to the Department of Conservative Dentistry and Endodontics, Hitkarini Dental College and Hospital, Jabalpur, with pain in his mandibular right second molar. Clinical examination revealed a deep carious lesion and the tooth was tender to percussion. There was absence of sinus tract. Vitality testing with a dry ice (R C ice Prime Dental) gave no response. A diagnosis of pulp necrosis with acute apical periodontitis was made and it was decided to go ahead with the endodontic treatment of the same tooth after informing the patient. After 2% lidocaine was administered the concerned area was isolated with a rubber dam (Hygenic, Coltene Whaledent). A conventional access cavity was prepared after excavation of caries. Clinical examination with a DG 16 (Hu-Freidy, USA) explorer revealed three mesial orifices and one distal canal (Figure 1). The canal lengths were measured using apex locator (ROOT ZX, MORITA). A radiograph was taken to confirm the working length and the presence of three mesial canals (mesiobuccal, mesiolingual, and middle mesial) and one distal canal (Figure 2). Three separate mesial orifices were verified using CBCT of the same tooth (Figure 3). The middle mesial canal joined apically with the mesiolingual canal. The canal orifices were widened using Gates Glidden drills (MANI) and apical preparation up to 30 no (2%) was carried out in the mesial canals and up to 35 no (2%) in the distal canal using K-Flexofiles (Dentsply Maillefer) and EDTA (Maillefer Dentsply, USA) as lubricant. Irrigation was carried using normal saline and 3% sodium hypochlorite (Vishal Dental products, India). After drying the canals with paper points (Dentsply, India), master cones (Dentsply, India) were selected which were confirmed by radiographs (Figure 4). The canals were obturated using AH Plus (DeTrey/Dentsply, Germany) as sealer by lateral condensation technique. A temporary dressing (Cavit G,3M ESPE, Germany) was given and a radiograph was taken to confirm the obturation (Figure 5). The patient was recalled after a week and was found to be asymptomatic. A full coverage crown was later on placed after a permanent restoration. The patient has been followed up for two years and is asymptomatic (Figure 6).

## 3. Discussion

Most of the times clinicians usually perceive that a particular tooth will have a predetermined number of roots and root canals. However various studies have shown that deviation from the normal is very frequent than earlier observed. Focus on higher magnification and better diagnostic aids like CBCT have improved clinical chances of diagnosing, locating, and treating extra canals.

Digital radiography has many advantages compared to conventional radiography like less radiation exposure, faster image acquisition without requirement of chemicals, and a number of processing tools such as magnification [8]. Computed tomography (CT) uses a fan shaped beam and multiple exposure around an object to reveal internal structure of an object [9]. They were reported for endodontic applications by Tachibana and Matsumoto in 1990 [10]. They had limited use in endodontics because of inadequate image detail and high cost. Cone beam computed tomography uses a cone shaped beam instead of the regular fan shaped one.

Matherne et al. in their in vitro study of 72 extracted teeth investigated the use of cone beam computed tomography (CBCT) as a diagnostic tool for identifying root canal systems and comparing them with images obtained by using charged couple device (CCD) and photostimulable phosphor plate (PSP). They concluded that when compared with CBCT, endodontists failed to identify one or more root canals in 4% of the teeth with CCD and 40% of the teeth with PSP [11].

Pomeranz et al. in their study of 100 molars (61 first and 39 second molars) reported 12 cases of middle mesial canals. Five of these were in second molars. They classified middle mesial canal into three categories: (1) fin, when at any stage of debridement the instrument could pass freely between mesiobuccal or mesiolingual canal and the middle mesial canal, (2) confluent, when the prepared canal originated as a separate orifice but apically joined the mesiobuccal or mesiolingual canal, and (3) independent, when the prepared canal originated as a separate orifice and terminated as a separate foramen [12].

Ahmed et al. in their study by clearing technique found the prevalence of three mesial canals in 4% of mandibular first molars and 10% in mandibular second molars of Sudanese population [13]. Aminsobhani et al. treated 27 mandibular molars with three mesial canals of which 21 teeth (77.8%) were first molars and 6 teeth (22.2%) were second molars. Two orifices, 3 root canals, and 2 apical foramina were seen in 2 cases. Three orifices and 2 apical foramina were seen in 14 cases. Three orifices and 1 apical foramen in 4 cases and 3 orifices, 2 root canals, 1 apical foramen were seen in 7 cases [14]. Beatty and Krell documented a mandibular first molar and a mandibular second molar with five canals. In both these cases mesial root had 3 canals and distal root had 2 canals [15].

Although a lot of authors have agreed on the presence of three foramina in the mesial root, very few have reported presence of three independent canals [16]. CBCT has been very successfully used in endodontics for a better understanding of root canal anatomy and evaluation of root canal preparation and vertical fractures. Robinson et al. evaluated mandibular first premolars on 120 routine dental CT images for variations in root and root canal system morphology. They identified 2 root canal systems in 16 mandibular first premolars. Panoramic evaluation of these same teeth demonstrated that 5 of these teeth appeared uniformly radiopaque at all root levels suggesting only one root canal system [17].

## 4. Conclusion

In this case report we confirmed the presence of three mesial canals in mandibular second molar with the aid of cone beam computed tomography (CBCT) which should be used as an adjunct for confirming the presence of complicated root canal anatomy, especially in situations where conventional periapical radiographs are not very conclusive.
